# MiR-7-5p inhibits thyroid cell proliferation by targeting the EGFR/MAPK and IRS2/PI3K signaling pathways

**DOI:** 10.18632/oncotarget.28030

**Published:** 2021-08-03

**Authors:** Alice Augenlicht, Manuel Saiselet, Myriam Decaussin-Petrucci, Guy Andry, Jacques E. Dumont, Carine Maenhaut

**Affiliations:** ^1^Institute of Interdisciplinary Research, Université libre de Bruxelles, Brussels, Belgium; ^2^Service d’Anatomie et Cytologie Pathologiques, Centre Hospitalier Lyon Sud, Université Lyon 1, Pierre Benite Cedex 69495, France; ^3^Surgery Department, J. Bordet Institute, Brussels 1000, Belgium

**Keywords:** miRNA, papillary thyroid carcinoma, IRS2, MAPK, PI3K

## Abstract

The aberrant expression of miRNAs is often correlated to tumor development. MiR-7-5p is a recently discovered downregulated miRNA in thyroid papillary carcinoma (PTC). The goal of this project was to characterize its functional role in thyroid tumorigenesis and to identify the targeted modulated pathways. MiR-7-5p overexpression following transfection in TPC1 and HT-ori3 cells decreased proliferation of the two thyroid cell lines. Analysis of global transcriptome modifications showed that miR-7-5p inhibits thyroid cell proliferation by modulating the MAPK and PI3K signaling pathways which are both necessary for normal thyroid proliferation and play central roles in PTC tumorigenesis. Several effectors of these pathways are indeed targets of miR-7-5p, among which EGFR and IRS2, two upstream activators. We confirmed the upregulation of IRS2 and EGFR in human PTC and showed the existence of a negative correlation between the decreased expression of miR-7-5p and the increased expression of IRS2 or EGFR. Our results thus support a tumor-suppressor activity of miR-7-5p. The decreased expression of miR-7-5p during PTC tumorigenesis might give the cells a proliferative advantage and delivery of miR-7-5p may represent an innovative approach for therapy.

## INTRODUCTION

MiRNAs are small endogenous single-strand RNA molecules of ~23 nucleotides in length, described as essential players in the regulation of gene expression by targeting coding mRNAs. Each miRNA can regulate the expression of more than several hundred genes by binding to the 3’UTR untranslated region of their target based on complementary sequences, blocking translation or causing the degradation of target mRNAs. All the theoretical targets of a miRNA can be defined *in silico* by using bioinformatic prediction models [1]. Because of their global cellular role, miRNAs are involved in a large amount of biological processes but also in many human diseases including cancer. Several studies have shown that many miRNAs are overexpressed or downregulated in tumor tissues compared to adjacent normal tissues [2, 3], and some evidence support their incidence in tumor initiation and/or progression [4–7].

Thyroid cancer is the most prevalent endocrine cancer by constituting almost 93% of cancers associated to this system [8, 9]. During the past decade, the incidence of thyroid cancers has been reported to increase by over 5% annually [10]. Papillary thyroid cancer (PTC) corresponds to approximately 85% of malignant thyroid cancers. Until a few years ago, PTC was characterized as a cancer presenting essentially upregulated miRNAs although in multiple human cancers a general downregulation of miRNAs is generally observed [11–15]. Nevertheless, with the emergence of the Next Generation Sequencing tools, a lot of downregulated miRNAs associated to PTC have been reported [16–18].

In this study, we have investigated the functional role of miR-7-5p, a miRNA previously identified in our studies as downregulated in PTC, and whose expression is associated with the presence of the BRAF^V600E^ mutation [17]. MiR-7-5p has a complex activity with different publications underlying the key role of this miRNA in various biological processes and/or human diseases; it has been reported to be either an oncogene or a tumor suppressor according to the cellular context. Indeed, miR-7-5p was shown to inhibit proliferation in lung carcinoma and glioblastoma, to inhibit migration and invasion in melanoma, and to promote proliferation and migration in lung carcinoma, skin epithelial cells and renal cancer [19–29]. MiR-7-5p can arise from three different loci and is highly conserved, supporting its key role [30–33]. An active role of this miRNAs in PTC tumorigenesis is thereby conceivable. However, the functional significance of its downregulation in PTC has not yet been investigated.

In this work, we have defined its role in different cellular processes by performing functional assays in miR-7-5p transfected thyroid cells and by identifying the modulated pathways. The *in vivo* relevance of our results was then addressed in PTC. Our data suggest that miR-7-5p inhibits thyroid cell proliferation by targeting the MAPK and PI3K signaling pathways.

## RESULTS

### MiR-7-5p is downregulated in papillary thyroid carcinomas and in thyroid derived cell lines

**Figure 1:**
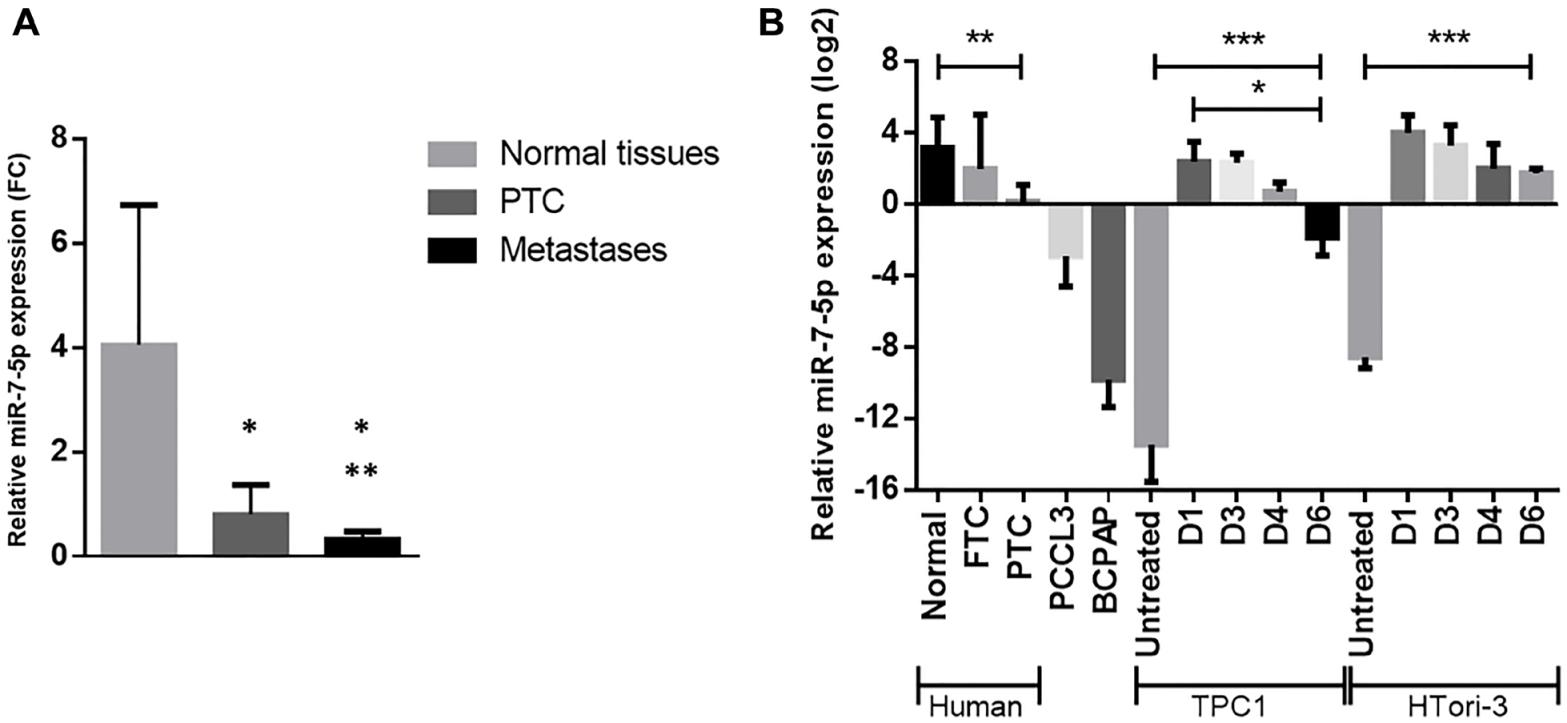
Downregulation of miR-7-5p in PTC and in thyroid derived cell lines, measured by RT-qPCR. (**A**) Experimental RT-qPCR validation in a set of 14 independent patient samples including normal adjacent tissues (black), PTC (blue), and associated lymph node metastases (red). Paired *t*-test was used to define statistically significant modulation (*p* < 0.05) of expression between normal samples and tumors or metastases (^*^) or between tumors and metastases (^**^)(adapted from Saiselet et al. 2016). (**B**) Relative miR-7-5p expression in human normal thyroid tissues (*n* = 14), FTC (*n* = 5) and PTC (*n* = 9), in various thyroid cell lines, and in TPC1 and HTori-3 cell lines on days 1, 3, 4 and 6 following miR-7-5p mimics transfection with *n* ≥ 4. Results are expressed as means with standard deviation. ANOVA test was used to define statistically significant modulation; ^*^ *p* < 0.05, ^**^ *p* < 0.01, ^***^ *p* < 0.001.

MiRSeq Deep Sequencing data, showing a strong downregulation of miR-7-5p, have been confirmed by RT-qPCR on 14 triplets of independent samples including PTC, normal adjacent tissues and associated lymph node metastases [17]. MiR-7-5p is downregulated in PTC samples compared to normal tissues and its expression is even significantly more downregulated in lymph node metastases (Figure 1A). Its expression was then assessed in different thyroid cell lines. TPC1 and HTori-3 cell lines were chosen among others for the functional studies for their low (Htori-3) or almost complete loss (TPC1) of endogenous expression of miR-7-5p (Figure 1B). To investigate its functional role, miR-7-5p has been transiently transfected in both cell lines. An increased expression of miR-7-5p was still observed 6 days after transfection (Figure 1B).

### MiR-7-5p inhibits thyroid cell proliferation, but has no impact on migration or invasion

**Figure 2:**
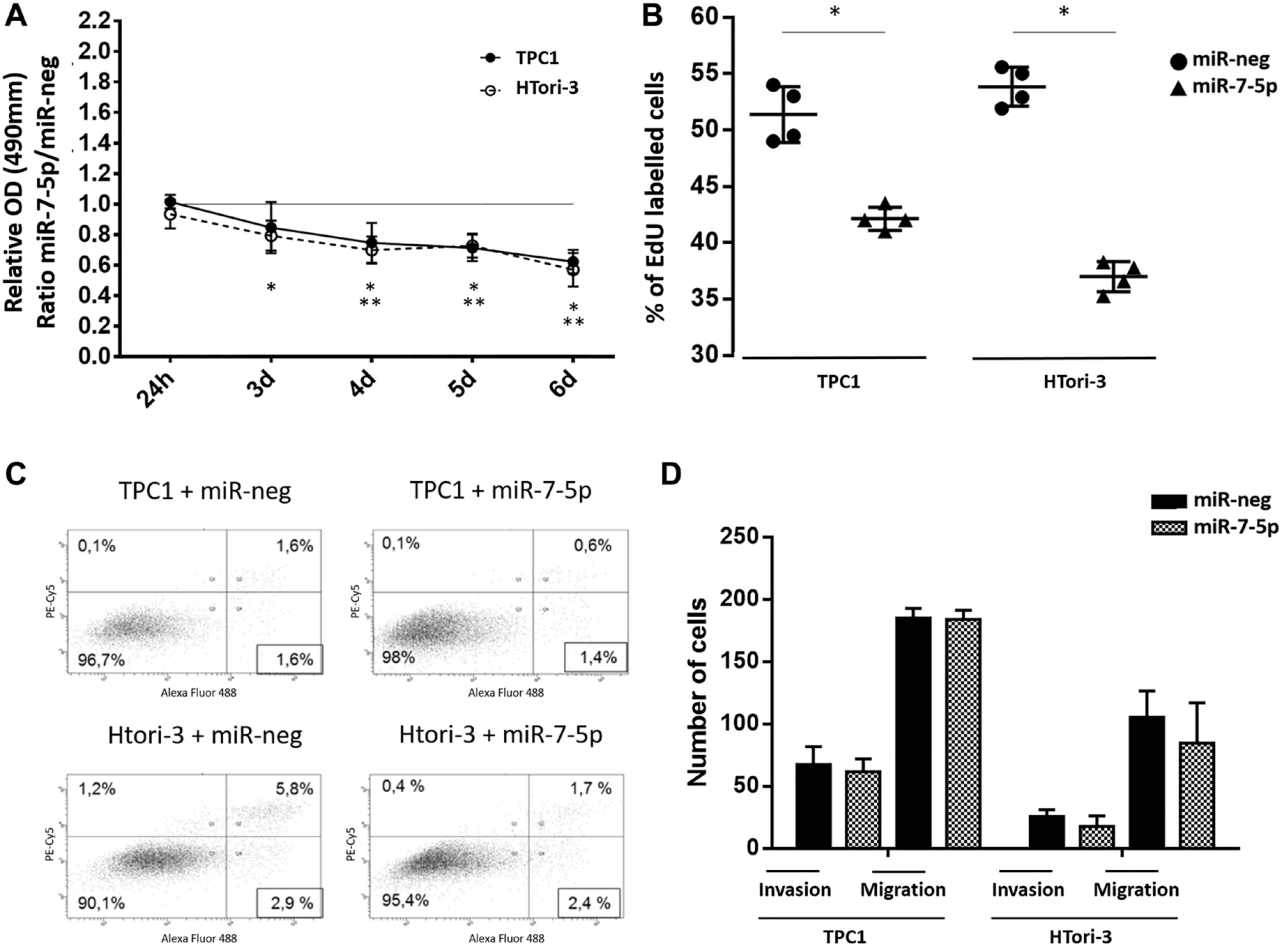
Functionnal consequences of miR-7-5p transfection in thyroid cell lines. (**A**) Ratio of absorbance of MTS metabolized reagent in miR-7-5p and miR-neg transfected cells. Paired *t*-test was used to define statistically significant modulation (*p* < 0.05) of expression between conditions in HTori-3 (^*^) or TPC1 (^**^) cells. (**B**) Percentage of EdU-positive cells among 20.000 cells analyzed by flow cytometry (*n* = 4). Results are expressed as means with standard deviation. Paired *t*-test was used to define statistically significant modulation (*p* < 0.05) between miR-7-5p transfected cells and the corresponding miR-neg group in TPC1 and Htori-3 cells. (**C**) 20.000 cells were analyzed by flow cytometry after labelling by SYTOX Advanced (X-Axis) to detect necrotic cells and by alexa 488-coupled Annexin V (Y-Axis) to detect apoptotic cells. Representative experiment with *n* = 3. (**D**) Counting and averaging of 5 separate spots of miR-7-5p or miR-neg transfected cells on coated matrigel chamber for invasion and on non coated chamber for migration (*n* = 4).

We have investigated the potential effect of miR-7-5p on cell growth [viability, proliferation and apoptosis]. Transfection of miR-7-5p led to a significant decrease of the proliferation rate in TPC1 and HTori-3 cells compared to cells transfected with the negative control, as determined by both MTS and EdU assays (Figure 2A and 2B). No effect on apoptosis was observed (Figure 2C), suggesting that the negative action of miR-7-5p on cellular growth results only from an inhibition of cell proliferation. A possible role of miR-7-5p on cell motility was then considered, using cell migration and invasion assays (Figure 2D). No effect was observed after miR-7-5p transfection in both cell lines, suggesting that miR-7-5p is not involved in these processes.

### Microarray analysis reveals a large number of deregulated genes following miR-7-5p transfection

In order to study more in depth the role of miR-7-5p in thyroid tumorigenesis, we performed a microarray analysis on miR-7-5p transfected cells. This allowed us to study the effect of miR-7-5p on the whole transcriptome instead of focusing on a few targets. Samples were harvested 3 days after transfection and hybridized onto Affymetrix Human Genome U133 Plus 2.0 arrays. The mRNA expression profiles of miR-7-5p transfected HTori-3 and TPC1 cells were normalized with their respective negative controls. The amount of statistically significant downregulated and upregulated genes following miR-7-5p transfection in each cell line is presented in Supplementary Table 1. We then looked into our gene list for the downregulation of validated targets of miR-7-5p. For that, we screened database of validated miRNA:mRNA interactions (miRTarBase) and recent literature to obtain the most complete list of validated mRNAs targets by a luciferase reporter assay of miR-7-5p (Supplementary Figure 1).

We have performed a Pathway Analysis using the David software (Database for Annotation, Visualization and Integrated Discovery) to reveal the enriched pathways after miR-7-5p transfection. Kegg pathway outputs were listed in Supplementary Table 2. In view of the large number of genes showing deregulation following miR-7-5p transfection, we decided to refine our analysis by minimizing dependence on the experimental model used. Hence, we only focused on genes commonly deregulated in the two cell lines, the 1054 genes commonly deregulated with a minimum fold change of 1.5 (up- or downregulated).

**Figure 3:**
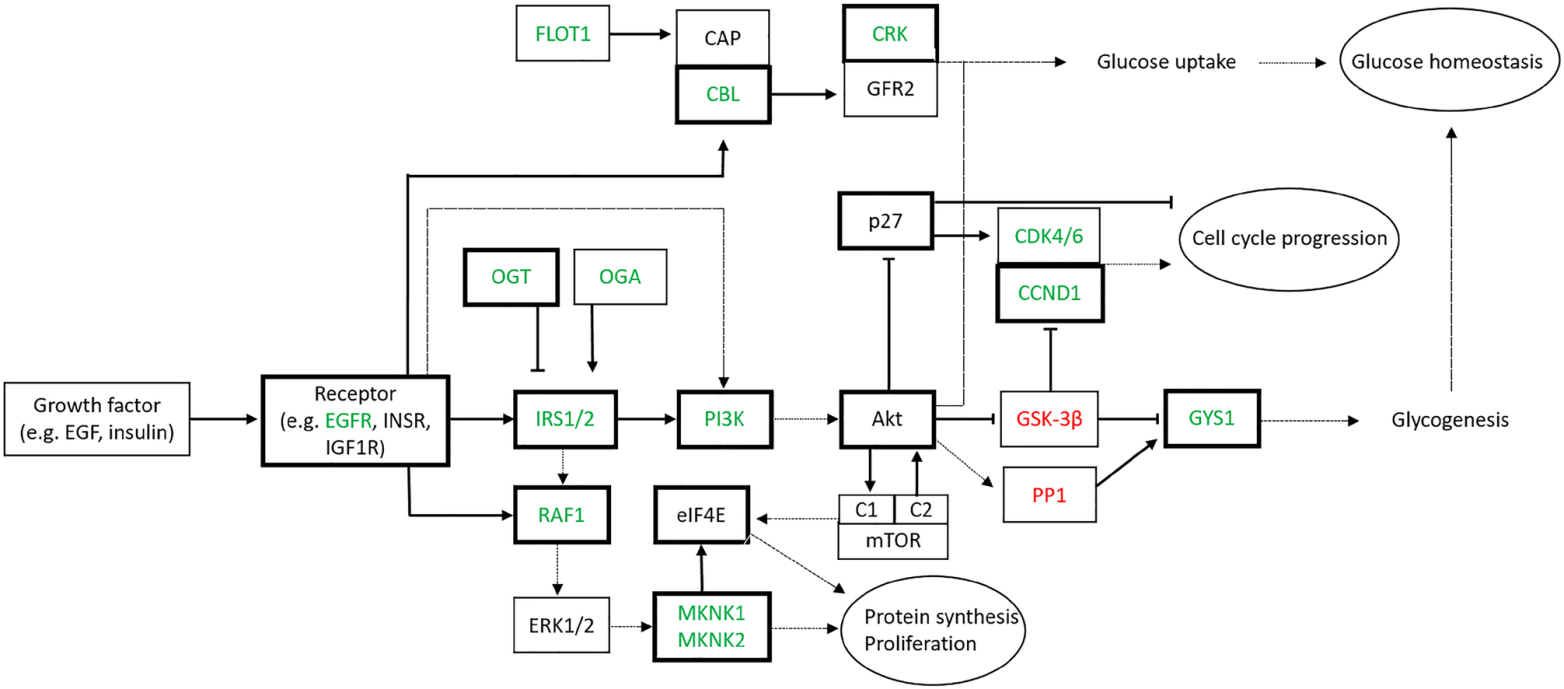
Signaling pathways targeted by miR-7-5p in thyroid cell lines. Genes identified by the Affymetrix microarray analysis with a fold change ≥ 1.5 are depicted (green: downregulated, red:upregulated). Arrows and bars indicate respectively stimulation and inhibition activity of the corresponding protein. Already validated targets of miR-7-5p are bold contoured (Abbreviations: EGFR: EGF Receptor; INSR: Insulin Receptor; IGF1R: IGF1 Receptor; IRS: Insulin Receptor Substrate; OGT: O-GlcNAc Transferase; OGA: O-GlcNAcase).

Three important pathways, known to be involved in the proliferation of thyrocytes, insulin signaling pathway, MAPK and PI3K-Akt signaling pathways were identified. The main actors involved in these pathways are synthetized in Figure 3. Interestingly, many components of these pathways are either validated or predicted targets of miR-7-5p, and are modulated accordingly. These data suggest that miR-7-5p inhibits the proliferation of thyroid cells via the EGF and insulin receptor signaling pathways, namely via the downregulation of the Insulin Receptor Substrate 2 (IRS2).

### Both MAPK and PI3K signaling pathways are inhibited by miR-7-5p

**Figure 4:**
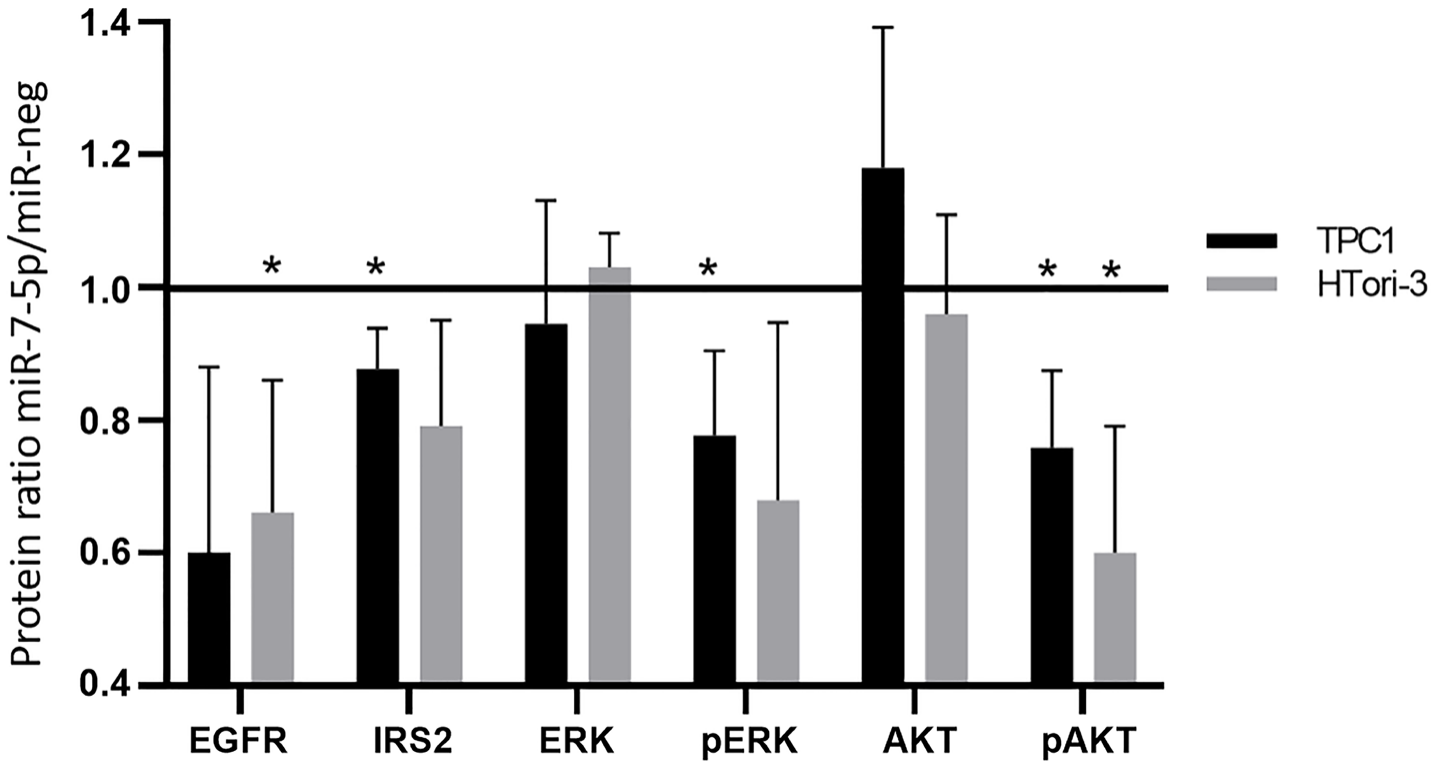
Protein expression and phosphorylation following miR-7-5p transfection in TPC1 and HTori-3 cells. Actin or vinculin were used as a loading controls. Relative protein expression between miR-7-5p condition and negative control. Results are expressed as means with standard deviation with *n* = 3. Paired *t*-test was used to define statistically significant modulation; ^*^ *p* < 0.05.

In order to further validate the microarray results, we selected several actors of the identified pathways and analyzed their protein expression and/or activity level by Western blotting (Figure 4). We observed similar decrease of mRNA and protein expression after miR-7-5p transfection in TPC1 and HTori-3 cells for EGFR and IRS2, two upstream activators of the MAPK and PI3K signaling pathways. To analyze the activity of these pathways, we measured the phosphorylation of ERK and AKT (Ser473). The decrease of the phosphorylation state of these proteins observed after miR-7-5p transfection, while protein expression is unchanged, supports the inhibitory role of miR-7-5p in the regulation of the MAPK and PI3K signaling pathways.

**Figure 5:**
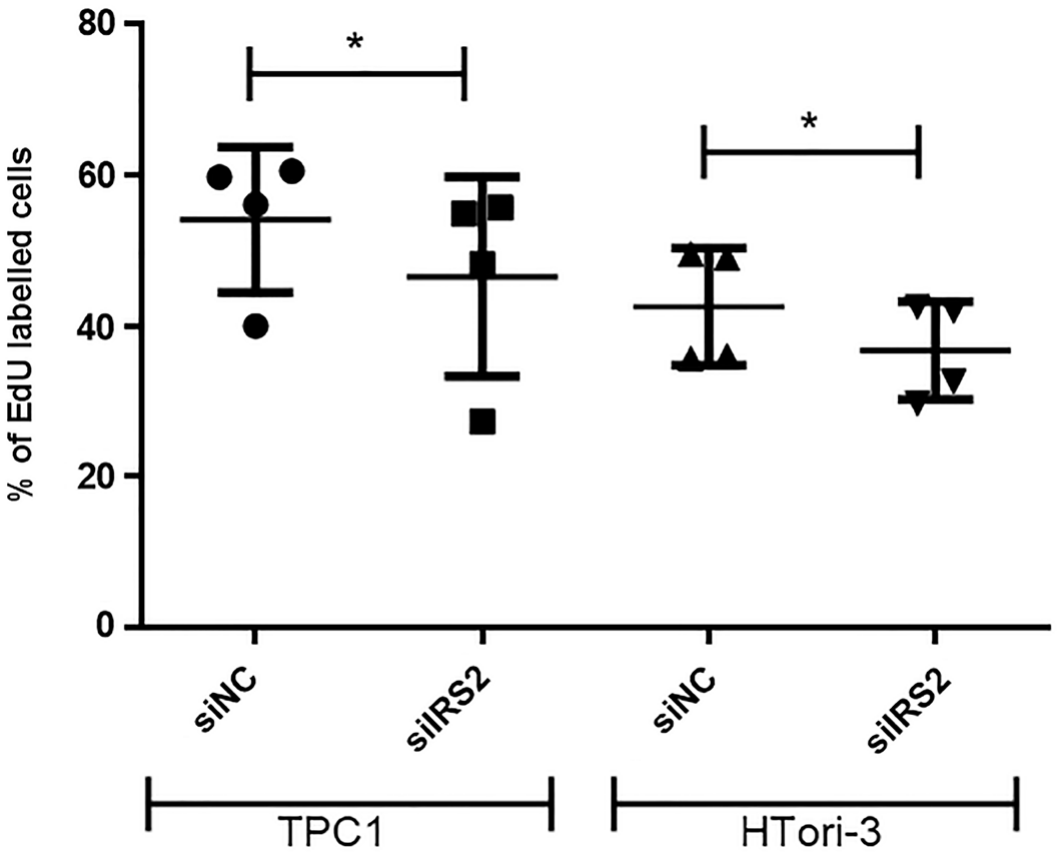
Inhibition of cell proliferation following IRS2 knockdown in TPC1 and HTori-3 cells. Percentage of EdU-positive cells among 20.000 cells analyzed by flow cytometry (*n* = 4) 3 days after transfection. Results are expressed as means with standard deviation. Paired *t*-test was used to define statistically significant modulation (*p* < 0.05) between siIRS2 transfected cells and the corresponding siNC group in TPC1 and HTori-3 cells.

**Figure 6:**
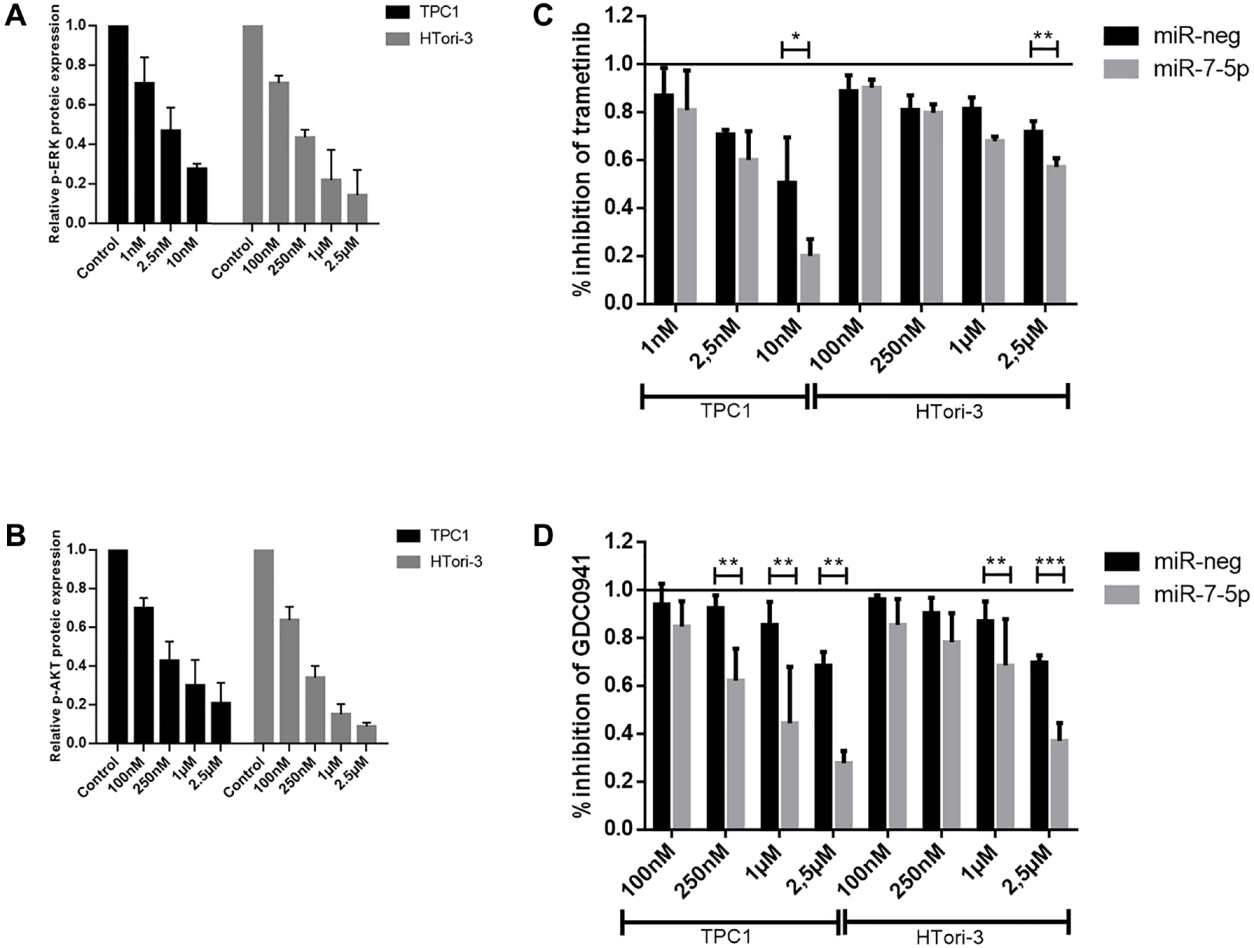
Cell proliferation after treatments with trametinib and GDC0941. Relative p-ERK and p-AKT protein expression after treatment with respectively trametinib (**A**) and GDC0941 (**B**) inhibitors at various concentrations. Control treatment condition consisted in DMSO-treated cells. For each condition after miR-7-5p or miR-neg transfection, a percentage of inhibition was defined after trametinib (**C**) and GDC0941 (**D**) treatment by normalizing the percentage of EdU labelled cells with the respective DMSO negative control treatment. Results are expressed as means with standard deviation (*n* ≥ 3). Paired *t*-test was used to define statistically significant modulation between miR-7-5p transfected cells and the corresponding miR-neg group in TPC1 and Htori-3 cells; ^*^ *p* < 0.05, ^**^ *p* < 0.01 and ^***^ *p* < 0.001.

Although there was only a slight decrease in IRS2 expression in our cell lines, we decided to focus on this protein and its potential regulation by miR-7-5p because IRS2 is an upstream signaling molecule that mediates the effects of insulin and whose regulation is linked to the insulin signaling pathway and the regulation of glucose homeostasis, two highlighted pathways in our DAVID analysis. To further establish whether miR-7-5p could mediate thyroid cell proliferation by regulating IRS2, we performed a siRNA knockdown of IRS2 in both cell lines. Proliferation assays showed that the decrease of IRS2 levels led to a reduction in the percentage of EdU labelled cells (Figure 5), suggesting that the inhibition of thyroid proliferation by miR-7-5p is at least partly mediated by IRS2. Additionally, we have evaluated the sensitivity of miR-7-5p transfected cells to the MEK inhibitor trametinib and to the PI3K inhibitor GDC0941. Various inhibitor concentrations were chosen for each cell line based on pERK and pAKT protein levels (Figure 6A and 6B) to assess the dose-dependent effect of trametinib and GDC0941 on thyroid cell proliferation, evaluated by EdU incorporation (Figure 6C and 6D). Both trametinib and GDC0941 inhibited proliferation in the TPC1 and Htori-3 cell lines. Moreover, transfection of miR-7-5p increased the sensitivity of the cells to the effects of these inhibitors. From these data, we concluded that the effects of miR-7-5p on thyroid cell proliferation are dependent on both MAPK and PI3K signaling pathways.

### MiR-7-5p and EGFR or IRS2 expressions are negatively correlated in human PTC

**Figure 7:**
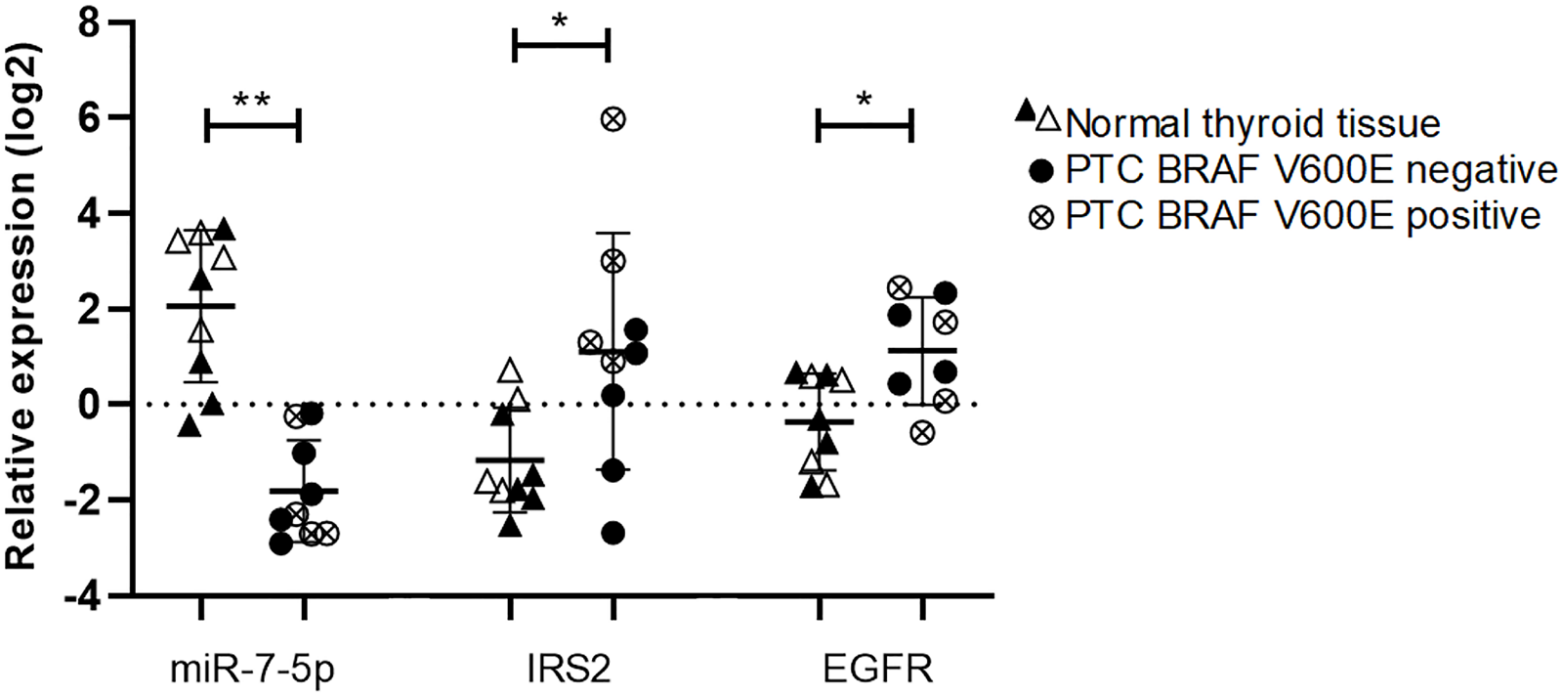
Dereregulation of miR-7-5p, IRS2 and EGFR measured by RT-qPCR. Relative expression in a set of 9 patient samples including normal adjacent tissues (triangles) and PTC (circles). BRAF^V600E^ mutation status was evaluated and used to separate BRAF^V600E^ negative (black) and positive (white) PTC. Presence of BRAF^V600E^ mutation is indicated by a cross and is absent in their matched normal thyroid tissues. Results are expressed as means with standard deviation. Paired *t*-test was used to define statistically significant modulation of expression between normal samples and tumors; ^*^ *p* < 0.05 and ^**^ *p* < 0.01.

**Figure 8:**
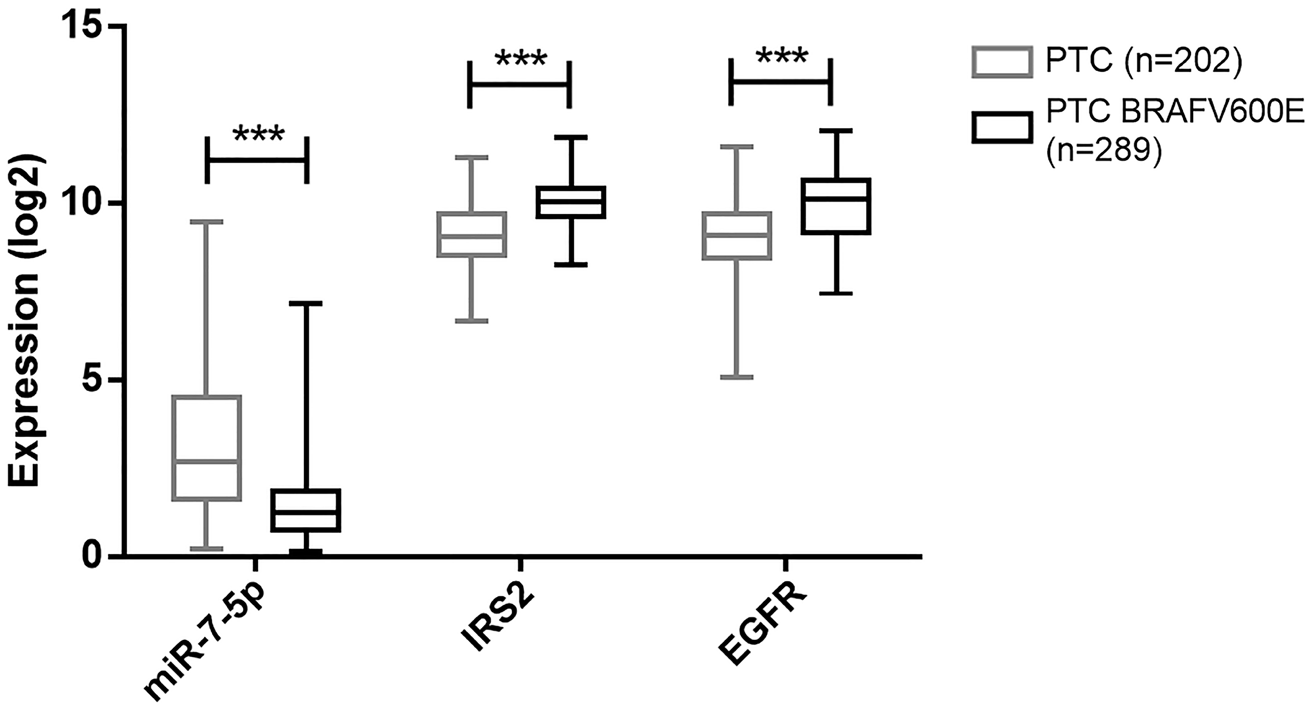
Dereregulation of miR-7-5p, IRS2 and EGFR according to PTC aggressivity. THCA study from TCGA database analyses of miR-7-5p, IRS2 and EGFR expression in BRAF^V600E^ negative (grey) and positive (black) PTC. Results are expressed as min-to-max box plots. Mann-Whitney test was used to define statistically significant modulation; ^***^ *p* < 0.001.

The EGFR and IGF-1R (insulin-like growth factor 1 receptor) signaling pathways play a central role in thyroid cell proliferation [34]. To address the *in vivo* relevance of our data, we quantified miR-7-5p, IRS2 and EGFR in 9 PTC and adjacent normal thyroids. Tumor samples showed a statistically significant downregulation of miR-7-5p and upregulation of IRS2 and EGFR, compared to their matched normal tissues (Figure 7). Since we showed previously that the downregulation of miR-7-5p was more pronounced in more aggressive PTC exhibiting a BRAF^V600E^ mutation [17], we hypothesized that IRS2 and EGFR expressions would be more increased in these BRAF^V600E^ positive PTC. When analyzing the TCGA, in addition to the significant downregulation of miR-7-5p, we indeed observed a significant upregulation of IRS2 and EGFR in BRAF^V600E^ positive compared to BRAF^V600E^ negative PTC (Figure 8). In our 9 PTC and matched normal thyroids, we observed a similar tendency despite the no robustness of the statistical analyses due to the low number of samples (Figure 7). Next, we evaluated the correlations between the decreased expression of miR-7-5p and the increased expression of IRS2 or EGFR (Table 1). In the TCGA data, expressions revealed a negative correlation between miR-7-5p and IRS2 (r_s_: –0.33, *p* = 1.73e-15) or EGFR (r_s_: –0.22, *p* = 2.55e-07) expressions, and a positive correlation between IRS2 and EGFR expressions (r_s_: 0.49, *p* = 4.54e-36). For the 9 pairs of PTC/normal thyroids, we could observe similar trends, although not statistically significant due to the small number of samples. These similar observations in our *in vitro* experiments and in human PTC support our hypothesis that miR-7-5p modulates thyroid cell proliferation by regulating different signaling pathways including MAPK, PI3K, and insulin signaling pathway with IRS2 as central actor.

**Table 1:** Correlations between miR-7-5p, IRS2 and EGFR mRNA expressions

Correlation	miR-7-5p vs IRS2		miR-7-5p vs EGFR		IRS2 vs EGFR	
	Spearman rho	*p* value	Spearman rho	*p* value	Spearman rho	*p* value
THCA study from TCGA database	–0,3291	1,73e-15	–0,2167	2,55e-07	0,4912	4,54e-36
9 paired couples Normal-Tumor	–0,717	0,037	–0,433	0,25	0,667	0,059

Correlations between miR-7-5p and IRS2, miR-7-5p and EGFR, IRS2 and EGFR expressions were evaluated from the PTC present in the TCGA database and from our experimental data on 9 pairs of PTC and adjacent normal tissues. Analyses from the TCGA database were performed on UCSC Xena browser and considered 580 samples from the THCA study including tumors (491), normal thyroid tissues (59) and metastases (9). For the 9 pairs of PTC and adjacent normal tissues analyzed by RT-qPCR, we considered the ratio of expression between tumor and normal tissues. These ratios were used to calculate the correlation of expression between the different genes.

## DISCUSSION

Since their discovery, miRNAs have been shown to regulate a lot of physiological processes and to be involved in various human pathologies including cancer, with a large amount of tumor suppressors and oncogenes targeted and regulated by miRNAs [11, 14, 35]. In a previous work whose purpose was to characterize the miRNA expression profile of PTC, several miRNAs have been identified as deregulated. Among these, miR-7-5p showed the most significant downregulation, what was confirmed by two other studies [16–18]. Nevertheless, little is known about its biological function and molecular mechanism of action in thyroid tumorigenesis. MiR-7-5p is described as oncomir or tumor suppressor according to the cellular context [19–29], although literature largely suggests that miR-7-5p acts as a tumor suppressor in most human tissues.

In the objective to characterize the functional consequences of the downregulation of miR-7-5p in PTC tumorigenesis, we have performed functional assays following miRNA transfection in thyroid derived cell lines to investigate proliferation, apoptosis and the ability of the cells to metastasize. Our results support an *in vitro* tumor-suppressor activity of miR-7-5p which inhibits cellular proliferation but has no impact on invasion or migration. Its decreased expression in human PTC could therefore play a role in the development and/or the progression of papillary thyroid cancer through the increase of cellular growth.

Our data are in accordance with previous studies showing similar results in glioblastoma and tongue carcinoma [19, 20, 36], but contrast with other studies reporting that miR-7-5p inhibits proliferation but also migration and invasion in thyroid cell lines [37–39]. It is likely that the differences result from the concentration of the transfected mimics which is 50 times higher in two studies, and not specified in the third. Indeed, Jin et al. reported that a high concentration of mimics induces non-specific alterations in gene expression [40], and in our study, we have observed a functional effect on survival, proliferation and migration with the negative control miRNA when transfected at a concentration of 20nM. Consequently, when used at supra-physiological concentrations such as in the published studies [37–39], an off-target effect is highly conceivable.

An innovative approach used in this work consists in defining the global biological effects –direct and indirect– of miR-7-5p in thyroid cell lines, by performing gene expression analysis. Indeed, the role of miRNAs as tumor suppressors or oncogenes is defined by the set of their biological targets [14, 35]. Hence, full characterization of the functional role of a given miRNA requires the validation of several direct targets and a correlation with the functional observations [15]. Since each miRNA has a large amount of mRNA targets, any change in miRNA expression may lead to many slight changes in all their targeted mRNAs, suggesting that the cellular reality is more complex than a linear control model [one miRNA/one mRNA target/one biological effect] as reported in most studies. We have confronted our mRNA expression data with the list of validated mRNA targets of miR-7-5p described in databases and in the literature. Only several of them were statistically downregulated after miRNA transfection in both cell lines. Interestingly, a lot of modulated mRNAs in our miR-7-5p transfected cells were already known to be targeted by miR-7-5p in thyroid or other tissues, including EGFR, IGF1R, IRS1, IRS2, RAF1, PI3K, MNK1, MNK2 (Figure 3) [19–21, 36, 41–45]. Our approach thus allowed us to get an enlarged and precise vision of the regulation exerted by miR-7-5p in a thyroid context.

Our Affymetrix gene expression analysis suggests that miR-7-5p inhibits cell proliferation by downregulating several members of the EGF and the insulin signaling pathways, among which EGFR and IRS2, an adaptor that mediates the effects of insulin and IGF-1. EGF is a well-known mitogenic agent for thyrocytes [34]. However, its mitogenic effect requires IGF-1 receptor stimulation by IGF-1 or by high concentrations of insulin, which have little mitogenic effects by themselves [34, 46]. EGF promotes a sustained activation of MAPK/ERKs, but only a weak and transient activation of the PI3K pathway, whereas insulin/IGF-1 strongly and sustainably activates the PI3K pathway, and weakly the MAPK/ERKs pathway [47]. This occurs following IRS2 binding to the receptor and its subsequent association with PI3K p85 subunit and GRB2 [48, 49]. To induce DNA synthesis, a strong and sustained activation of both pathways are necessary [47]. We have experimentally validated that the decrease of IRS2 in the two thyroid cell lines led to a reduction of cell proliferation, supporting its role in the regulation of thyroid proliferation. In accordance with our results, an increase of circulating levels of insulin is correlated to an increase of thyroid proliferation [50, 51], and increased expression of insulin receptors has been described in cancers, including thyroid carcinomas [52, 53]. Our data show that miR-7-5p negatively controls thyroid cell proliferation by downregulating actors in both pathways, and the decrease of MAPK and PI3K signaling activity following miR-7-5p transfection supports the dependence of these pathways in miR-7-5p function.

To further address the *in vivo* relevance of our results, we investigated the relationships between miR-7-5p, IRS2 and EGFR expressions in PTC. Tumor samples showed a downregulation of miR-7-5p and conversely an upregulation of IRS2 and EGFR. More interestingly, when analyzing the TCGA data, a negative correlation between miR-7-5p and IRS2 or EGFR expressions was observed. MiR-7-5p, IRS2 and EGFR expressions are correlated with the aggressivity of thyroid tumors: more aggressive BRAF^V600E^ positive PTC exhibit a more pronounced decrease of miR-7-5p expression and increase of IRS2 or EGFR expressions compared to BRAF^V600E^ negative PTC. This suggests that the relationship between miR-7-5p, IRS2 and EGFR may play a significant role in thyroid tumorigenesis. In adequacy with our findings, other studies showed similar results by suggesting an anti-proliferative role of miR-7-5p through targeting the EGFR, MAPK and IGF1R/Akt signaling pathways in different tumoral contexts, such as glioblastoma and tongue carcinoma [19, 20, 36].

Our data suggest that glucose homeostasis is deregulated following miR-7-5p transfection. This miRNA has been largely studied in the endocrine pancreas because it is the most abundant miRNA in β-cell islets [54, 55]. MiR-7-5p regulates β-cell proliferation through a miR-7-mTOR axis [24, 56], driving β-cell mass, proliferation, survival and function [57]. Following miR-7-5p disruption in beta-cells, an increase of pAkt (Ser473), Mnk1/2 and Mapkap1 protein levels were reported [44], similarly to what we observed here in thyroid cells. In accordance, previous publications reported that PTC present an overactivation of the mTOR pathway and an increase of phospho-Akt Ser473 expression [58, 59].

Future studies should explore the mechanisms by which miR-7-5p is downregulated during thyroid tumorigenesis. MiR-7-5p can originate from 3 different loci whose transcription is independently regulated. While MIR-7-2 and 7-3 are intergenic, the first locus is in the last intron of the hnRNPk gene. Analysis of the TCGA revealed that hnRNPK is not modulated in PTC, suggesting that the downregulation of miR-7-5p cannot be explained by the decreased transcription of its host gene. Different transcription factors regulate miR-7-5p expression [31, 44, 60], among which NFκB, which inhibits miR-7-5p promoter activity of the MIR-7-1 and 2 loci in gastric cancer [33]. NFκB is activated following PI3K stimulation, and in addition, RELA and RELB mRNA expressions are significantly increased in the PTC from TCGA [61, 62]. Consequently, the loss of expression of miR-7-5p could be explained by the inhibition exerted by NFκB on the transcription of locus 1 and 2 of miR-7-5p, and this should be addressed in future studies.

In conclusion, we have evaluated the functional role of miR-7-5p in thyroid cells and shown that this miRNA negatively regulates the expression of several signaling targets active in the MAPK and PI3K/Akt signaling pathways. Hence, its decreased expression during PTC tumorigenesis could give the cells a proliferative advantage. Given the central roles of the MAPK and PI3K/Akt pathways in PTC tumorigenesis, delivery of miR-7-5p may represent an innovative approach for therapy, especially for high proliferative PTC recurrences. Future research on miR-7-5p will allow to define its potential therapeutic application by using already available delivery systems like cell-penetrating peptides, liposomes, micelles, and polymeric nanoparticles [63, 64].

## MATERIALS AND METHODS

### Thyroid tissues

Normal thyroid tissues, adjacent tumors (PTC, classical variant, and FTC) and PTC matched lymph node metastases (LNM) were collected in the Centre Hospitalier Lyon Sud (Lyon, France) and in the J. Bordet Institute (Brussels, Belgium). Protocols have been approved by the ethics committees of the institutions. Written informed consent was obtained from all participants involved in the study.

### Cell culture and miRNA transfection

The TPC1 cell line is derived from a human RET/PTC1-positive PTC and the Htori-3 cell line is derived from SV40-immortalized human thyrocytes. The TPC1 cell line was received from Pr. Mareel (University of Ghent, Belgium) and the HTori-3 cell line was received from Pr. Wynford-Thomas (Hammersmith Hospital, London, UK). Short tandem repeat profiling confirmed the thyroid identity of the cell lines [65]. Both cell lines were cultured at 37°C in 5% CO_2_ with RPMI 1640 (Invitrogen) supplemented with 10% fetal bovine serum. Cells were transiently transfected with miRIDIAN microRNA mimics miR-7-5p (#C-300546-07-0005) or negative control cel-miR-67 (#CN-001000-01-05) using DharmaFECT 2 (Dharmacon) and with siRNA IRS2 silencer select (#4392420) or negative control (#4390843) using RNAiMAX lipofectamine (ThermoFisher) according to the manufacturer’s protocol. Transfected miRNA mimics and negative control miRNA were used at a concentration of 2 nM while siRNAs and controls were used at a concentration of 20 nM. Cells were treated with trametinib and GDC0941 inhibitors for 24 h at various concentrations and analyzed 3 days post-transfection.

### RNA purification, reverse transcription and quantitative PCR

Total RNA extraction was performed using QIAzol reagent (Qiagen) and miRNeasy Mini kit (Qiagen). Total miRNA reverse transcription was performed with miRCURY LNA Universal RT microRNA PCR kit (Exiqon). Quantitative PCR was performed with cDNA Synthesis and SYBR Green Master Mix kit [Exiqon]. Primers used were LNA-enhanced oligonucleotides miR-7-5p (#205877, Exiqon). Relative expression was calculated using adapted Pfaffl method [66] with U6 snRNA (#308006, Exiqon) as endogenous control for normalization. All kits were used according to the manufacturer’s protocol.

### Cell viability and proliferation assays

Cell viability was evaluated by MTS assay (Promega). Cells were incubated with MTS reagents for 1 h 45 every 24-hours over a period of 6 days. Quantification was performed by measuring the absorbance at 490 nm. Cell proliferation was evaluated by 5-ethynyl-2′-deoxyuridine EdU incorporation assay (Click-iT EdU Flow Cytometry Assay Kit, Life Technologies). EdU reagent was used at a final concentration of 10 μM 6 h before harvesting the cells which were treated according to the manufacturer’s protocol. Cells were analyzed 3 days after transfection. EdU incorporation was analyzed by flow cytometry on the BD LSRFortessa cell analyzer.

### Apoptosis assay

Apoptosis was evaluated by Annexin V Conjugates assay (Invitrogen), according to the manufacturer’s instructions. Cells were analyzed 3 days after transfection.

### Migration and invasion assay

The invasive and migration abilities of the cells were analyzed by Transwell chamber assays with or without coated matrigel (Corning). 2.5 × 10^4^ TPC1 cells and 2 × 10^4^ HTori-3 cells were harvested 3 days after transfection with a solution of PBS/5 mM EDTA/5 mM EGTA. 22-hours after seeding into the upper chamber, passing cells were fixed, stained by using Diff-Quick stain kit (Polysciences) and quantified by counting and averaging 5 separate spots of 4 mm diameter.

### Affymetrix microarray studies

Cells were lysed 3 days after transfection with QIAzol reagent (Qiagen) and RNAs were extracted using RNeasy Mini kit (Qiagen). The RNA concentrations were spectrophotometrically quantified, and their quality was evaluated by the Experion Automated Electrophoresis System (Bio-rad). Samples were hybridized onto Affymetrix U133 plus 2.0 Arrays using 3’IVT Plus Labelling kit (AROS Applied Biotechnology A/S) according to the manufacturer’s protocol.

### Protein extraction and western blotting

Proteins were extracted 3 days after transfection. Detailed protocol and detection methods used were previously described [65]. Primary antibodies used were against EGFR (1:1000; #2232), AKT (1:1000; #9272) and p-AKT (1:1000; #9277S) from Cell Signaling Technology, IRS2 (1:100; #390761), ERK (1:500; #514302), p-ERK (1:500; #7383) and Vinculin (1:500; #25336) from Santa Cruz biotechnology and Actin (1:1000; #A2066) from Sigma. Secondary antibodies were anti-rabbit IgG HRP-linked (1:3750; #7074S) and anti-mouse IgG HRP-linked (1:3750; #7076S) from Cell Signaling technology.

### Bioinformatics analyses

The .cel files were processed using a preprocessed GenePattern program to create mRNA expression files and to perform a log2 transformation of the data. Normalization was performed based on the expression profiles of cells transfected with the negative control. mRNAs with a fold change ≥ 1.5 were selected. Pathway analyses were carried out with DAVID Bioinformatics Resources 6.7; the criteria for pathway selection in Kegg Pathway database were False Discovery Rate (FDR) ≤ 5% or False Discovery Rate (FDR) ≤ 7.5% and Fold Enrichment ≥ 1,5 with *p* < 0.05. Gene expression data are available from the Gene Expression Omnibus under accession number GSE159666.

## SUPPLEMENTARY MATERIALS


