# Supplementary material for: MiR-7-5p inhibits thyroid cell proliferation by targeting the EGFR/MAPK and IRS2/PI3K signaling pathways
Source: Oncotarget. 2021 Aug 3;12(16):1587–99. doi: 10.18632/oncotarget.28030 (PMC8351599; doi:10.18632/oncotarget.28030)
Supplement: Supplementary file 1 [file oncotarget-12-1587-s001.pdf]

MiR-7-5p inhibits thyroid cell proliferation by targeting the EGFR/MAPK and IRS2/PI3K signaling pathways

SUPPLEMENTARY MATERIALS

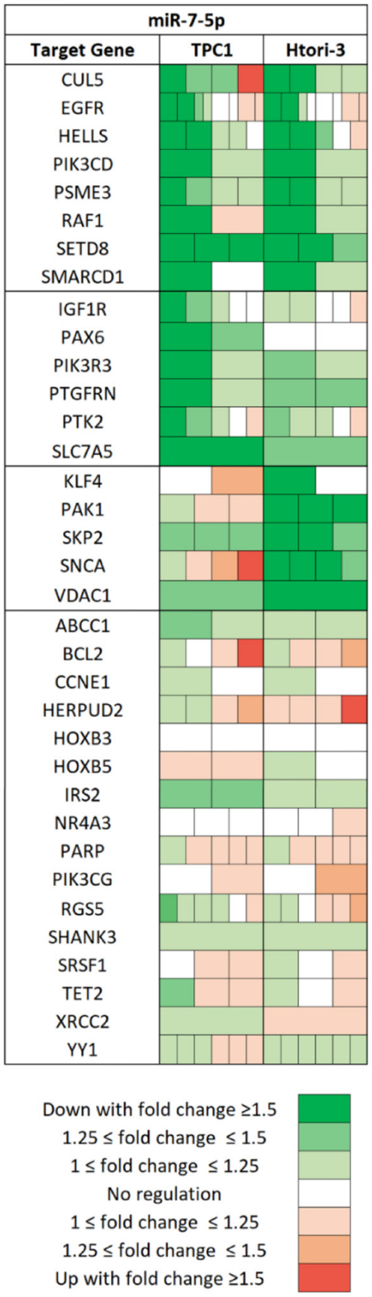

Supplementary Figure 1: List of targets of miR-7-5p validated by luciferase reporter assay and their corresponding expression in TPC1 and HTori-3 cells after miR-7-5p transfection. For each validated target of miR-7-5p, all the probes present on the array have been taken into account and are represented by a colored box. Fold change for each gene was defined after normalization by using gene expression ratios between miR-7-5p and miR-neg transfected cells. Each probe is associated with a color according to the amplitude of the fold change, as shown in the legend.

Supplementary Table 1: Affymetrix genes analysis

	TPC1	Htori-3	Common
**Fold change ≥1.5**			
Downregulated genes	1705	2037	488
All deregulated genes	3454	4095	1054
**Fold change ≥2**			
Downregulated genes	467	619	136
All deregulated genes	820	1101	195

Number of downregulated and deregulated genes with a fold change ≥1.5 or ≥2 following miR-7-5p transfection in TPC1 or Htori-3 cells. The third column represents the genes commonly down or deregulated in both cell lines.

Supplementary Table 2: Kegg pathway ontologies

TPC1			
**Term**	***P* Value**	**Fold Enrichment**	**FDR**
hsa04722:Neurotrophin signaling pathway	0,00002	2,2181	0,0053
hsa04151:PI3K-Akt signaling pathway	0,0001	1,5913	0,0087
hsa05212:Pancreatic cancer	0,0001	2,5594	0,0087
hsa05205:Proteoglycans in cancer	0,0002	1,7884	0,0087
hsa05210:Colorectal cancer	0,0002	2,5491	0,0087
hsa04668:TNF signaling pathway	0,0003	2,0989	0,0087
hsa04931:Insulin resistance	0,0003	2,0795	0,0087
hsa04510:Focal adhesion	0,0003	1,7363	0,0087
hsa05200:Pathways in cancer	0,0003	1,5027	0,0087
hsa04910:Insulin signaling pathway	0,0004	1,9288	0,0087
hsa05169:Epstein-Barr virus infection	0,0005	1,9772	0,0104
hsa04066:HIF-1 signaling pathway	0,0019	1,9929	0,0355
hsa04210:Apoptosis	0,0021	2,2807	0,0355
hsa05222:Small cell lung cancer	0,0021	2,0550	0,0355
hsa04068:FoxO signaling pathway	0,0022	1,8002	0,0355
hsa00310:Lysine degradation	0,0026	2,3994	0,0394
hsa05161:Hepatitis B	0,0036	1,7210	0,0522
hsa04550:Signaling pathways regulating pluripotency of stem cells	0,0042	1,7230	0,0560
hsa05100:Bacterial invasion of epithelial cells	0,0043	2,0262	0,0560
hsa05164:Influenza A	0,0047	1,6254	0,0585
hsa05142:Chagas disease (American trypanosomiasis)	0,0053	1,8396	0,0622
hsa04012:ErbB signaling pathway	0,0064	1,9122	0,0696
hsa04010:MAPK signaling pathway	0,0066	1,4795	0,0696
hsa04014:Ras signaling pathway	0,0070	1,5090	0,0696
hsa05166:HTLV-I infection	0,0071	1,4737	0,0696
hsa05215:Prostate cancer	0,0073	1,8905	0,0696
hsa04144:Endocytosis	0,0076	1,4841	0,0696
hsa00280:Valine, leucine and isoleucine degradation	0,0080	2,3007	0,0706
hsa04520:Adherens junction	0,0088	1,9916	0,0721
hsa05214:Glioma	0,0088	2,0475	0,0721

hsa05220:Chronic myeloid leukemia	0,0100	1,9640	0,0757
hsa05168:Herpes simplex infection	0,0103	1,5454	0,0757
hsa01100:Metabolic pathways	0,0104	1,1737	0,0757
hsa04152:AMPK signaling pathway	0,0104	1,6907	0,0757
hsa05221:Acute myeloid leukemia	0,0135	2,0795	0,0949
hsa04512:ECM-receptor interaction	0,0138	1,8166	0,0949

**Htori-3**			
**Term**	***P* Value**	**Fold Enrichment**	**FDR**
hsa04931:Insulin resistance	0,00001	2,2125	0,0034
hsa04115:p53 signaling pathway	0,0001	2,4857	0,0069
hsa04910:Insulin signaling pathway	0,0004	1,8365	0,0319
hsa04510:Focal adhesion	0,0005	1,6521	0,0319
hsa04144:Endocytosis	0,0009	1,5624	0,0485
hsa00514:Other types of O-glycan biosynthesis	0,0016	3,2914	0,0697
hsa04152:AMPK signaling pathway	0,0022	1,7661	0,0819
hsa04010:MAPK signaling pathway	0,0028	1,4883	0,0914
hsa04142:Lysosome	0,0035	1,7355	0,0968
hsa05100:Bacterial invasion of epithelial cells	0,0037	1,9495	0,0968
hsa04666:Fc gamma R-mediated phagocytosis	0,0041	1,8965	0,0980
hsa05212:Pancreatic cancer	0,0058	2,0052	0,1071
hsa04722:Neurotrophin signaling pathway	0,0061	1,6896	0,1071
hsa04071:Sphingolipid signaling pathway	0,0061	1,6896	0,1071
hsa05202:Transcriptional misregulation in cancer	0,0065	1,5609	0,1071
hsa05211:Renal cell carcinoma	0,0068	1,9748	0,1071
hsa05220:Chronic myeloid leukemia	0,0076	1,9108	0,1071
hsa05169:Epstein-Barr virus infection	0,0076	1,6619	0,1071
hsa04068:FoxO signaling pathway	0,0080	1,6211	0,1071
hsa04210:Apoptosis	0,0084	1,9854	0,1071
hsa05146:Amoebiasis	0,0086	1,7078	0,1071
hsa04014:Ras signaling pathway	0,0100	1,4418	0,1090
hsa04151:PI3K-Akt signaling pathway	0,0102	1,3433	0,1090
hsa05222:Small cell lung cancer	0,0103	1,7890	0,1090
hsa03460:Fanconi anemia pathway	0,0107	2,0494	0,1090
hsa04662:B cell receptor signaling pathway	0,0109	1,8890	0,1090

Kegg pathway analysis of deregulated genes in miR-7-5p transfected TPC1 (upper board) and Htori-3 (lower board) cell lines.
